# Repetitive head impacts affect mediolateral postural sway entropy in the absence of vision following a competitive athletic season: preliminary findings

**Published:** 2020-04-16

**Authors:** Brian Szekely, Sushma Alphonsa, Katelyn Grimes, Barry Munkasy, Thomas Buckley, Nicholas G. Murray

**Affiliations:** ^1^Department of Psychology; ^2^Department of School of Community Health Sciences, Division of Kinesiology, University of Nevada, Reno, 89557, Nevada; ^3^Department of Athletic, University of Richmond, 23173, Virginia; ^4^Department of Health Sciences and Kinesiology, Georgia Southern University, Statesboro, Georgia; ^5^Department of Kinesiology and Applied Physiology, University of Delaware, Newark, 19713, Delaware, United States

**Keywords:** center of pressure, regularity subconcussive impacts, balance, mTBI, sports

## Abstract

**Background:**

Repetitive head impacts (RHIs) have received more notice over the past decade. More sensitive measures, such as postural control, have been used to evaluate if there are biomechanical changes after RHI exposure. Similar to the clinical findings, most of the studies have failed to find any significant changes across an athletic season. However, these studies included those with a concussion history and only assessed postural control in the eyes open (EO) condition, rather than in both the EO and eyes closed (EC) conditions.

**Aim:**

The purpose of this study was to investigate postural control changes during quiet stance following a season of RHI in Division I football athletes who did not have a prior diagnosed signet ring cell compared to a group of non-RHI athletes with no history of a diagnosed sport-related concussion.

**Materials and Methods:**

Eighteen male Division I athletes were recruited and met the inclusion criteria: Nine football athletes (RHI group) and nine baseball athletes (CON group). All athletes performed three 30 s trials while standing with feet together on a force platform during EC and EO conditions. Center of pressure data was analyzed with sample entropy (SampEn) in the anteroposterior (AP) and mediolateral (ML) directions. SampEn data were analyzed with a three-level linear mixed effects model or the multilevel model, with the three levels being condition, time, and group.

**Results:**

The analysis reported no significant effect for SampEn AP, but reported a significant three-way interaction (Group by Task by Time) for SampEn ML. Specifically, SampEn ML was significantly higher for EC than EO for both groups.

**Conclusions:**

There are postural control changes from pre- to post-season, with the main contributor being EC postural control. Thus, there could be a change in the sensory reweighting dynamics due to RHI and the effect of sport.

**Relevance for patients:**

RHI may be better assessed in the clinical setting with EC, rather than with EO. Furthermore, clinicians should include tasks that deprive sensory inputs to examine the effects of RHI.

## 1. Introduction

Repetitive head impacts (RHIs) are a growing concern due to their possibility of causing alterations in brain function [1]. RHIs are operationally defined as multiple blows to the head and body that do not induce a clinical diagnosis of a concussion [2,3]. Prior research has indicated a relationship between the magnitude of head impact exposures and changes in white matter connectivity [4,5]. Furthermore, athletes with a high frequency of RHI also exhibited decreased activation of the dorsolateral prefrontal cortex, which is known to play a primary role in decision-making [6,7]. Interestingly, these changes in neuronal activity have shown to not affect cognitive function post-RHI exposure in high school and collegiate athletes [8-11]. However, clinicians still predominantly use clinical assessments to aid the effect of RHI exposure, such as the Sensory Organization Test, Graded Symptom Checklist, and Balance Error Scoring System [12].

Several clinical assessments have been used to evaluate the effect of RHI exposures. However, these assessments have failed to detect any clinically meaningful changes from pre- to post-collegiate season [12]. Therefore, more sensitive measures, such as changes in the postural control system obtained from force platforms, can help clinicians better understand the effect of RHI on athletic postural control. Thus far, the effect of RHI while maintaining an upright stance on a force platform has been sparsely evaluated. Only one prior study has reported increased postural sway velocity following an athletic season [13]. In addition, a few studies have reported that RHI does not affect static [12,14,15] or dynamic [16] postural control following an athletic season. Interestingly, all of these studies have only included participants that have had a history of concussion, which is known to increase postural control velocity, magnitude, and entropy values [17-19].

To address postural control as affected by RHI, prior literature has used entropy measures [14]. Specific entropy measures, such as sample entropy (SampEn), have been extensively used to quantify center of pressure complexity [20]. Low entropy values (or high regularity) are an indication of postural constraint and reduced adaptation to potential postural homeodynamic disturbances [21]. In the context of RHI and postural control, Murray *et al*. [14] reported no difference in regularity between RHI and control groups while they performed upright stance. However, a caveat to this study was that the history of concussion was not accounted for, which has been shown to decrease both anteroposterior (AP) and mediolateral (ML) postural control entropy [17-19]. In addition, lingering postural control abnormalities have been noted through entropy measures well beyond a year post-injury [14,22]. Thus, it is important to study how RHI may impact the postural control system. In addition to using those with a concussion history [14], only used eyes open (EO) static stance, rather than using both eyes closed (EC) and EO. The EO task indicates a visual orientation in space, which increases postural stability [21]. However, the EC task is controlled significantly by the vestibular and proprioceptive systems and decreases postural stability, which may be a more sensitive tool in pathologies [21,23,24]. Therefore, the purpose of this study was to investigate postural control changes during quiet stance following a season of RHI in Division I football athletes who did not have a prior diagnosed sport-related concussion compared to a group of non-RHI athletes with no history of a diagnosed sport related concussion. Specifically, we hypothesized that the RHI group would have similar entropy values in both AP and ML directions for the non-contact (CON) group during EO and EC quiet stance tasks.

## 2. Materials and Methods

### 2.1. Participants

Based on prior research [14,25], an a priori power analysis (alpha=0.05, power=0.80) determined that a total of nine participants per group were required to elicit a significant effect. Therefore, 18 male Division I athletes were recruited from a single Division I university: Nine football players (RHI; 20±2 years) were included in the RHI group and nine baseball players (CON; 20±1 years; Table 1) for the CON group. Table 1 describes participant demographics with means (M), standard deviations (SD), and *P*-values (*P*) for each measure. Baseball players were chosen as the control group due to the minimal risk of RHI. Participants from any group were excluded if they self-reported a medically diagnosed sport-related concussion or had a history of neurological, cognitive, or behavioral disorders. In addition, any participants that were withheld from athletic participation or had any altered gait due to any acute lower extremity injuries were also excluded. Of the 26 possible athletes, seven (four RHI and three CON) were excluded due to prior concussion history. Informed consent was obtained from all participants and the study was approved by the Institutional Review Board and Ethics Committee at the respective university (Decision number: H16114).

**Table 1:** Demographics of groups (CON and RHI) following inclusion and exclusion criteria.

Measure	CON	RHI	*P*
	
	M±SD	M±SD	
Age (years)	20.4±1.6	19.7±1.1	0.295
Height (cm)	184.7±4.6	183.1±3.3	0.409
Weight (kg)	81.1±29	81.8±5.4	0.944
Time between testing (days)	94±44.29	121.33±53.7	0.256

M: Mean, SD: Standard deviations

### 2.2. Procedures

Center of pressure data was collected at pre-participation physicals and at post-season (within 48 h of a season’s conclusion). Participants performed three 30 s trials of EC and EO feet together upright stance on a force platform (AMTI, Model OR-6, 1000 Hz, Watertown, MA, USA). The 30s trials were selected due to their high reliability in static postural tasks [21]. During the EC task, the data collection time did not start until 5 s after the participants closed their eyes to allow for postural adjustment settling [21]. Time of season was collected and calculated as when the first off-season occurred to the last athletic event of the season (Table ). Individual head impact data were not collected in the RHI group across the season or from prior seasons. However, the RHI group means±SDs for mean linear acceleration and number of head impacts were given from the Head Impact Telemetry System (HITS, 1000 Hz, Riddell, Chicago, IL. USA) sensors that were placed in the helmet of each RHI participant. The RHI group experienced a mean linear acceleration of 30.7±6.8 g over the course of 52 practice and 19 game/scrimmage sessions. The number of impacts recorded across all RHI participants was 2207 with 204 (9.2%) over 90 g.

### 2.3. Data analysis

Raw center of pressure data was recorded by the AMTI force plate and processed in Vicon Nexus software (Nexus, Version 1.8.5, Vicon Motion Systems, Oxford, UK) [17]. The raw center of pressure data was exported to MATLAB (MATLAB 2017a, Mathworks Inc., Natick, MA) and down sampled to 10 Hz with the resample function in MATLAB [26]. The downsampled data were not filtered for the entropy analyses as the filtering process is known to blunt or obliterate the most meaningful intricacies of the data [27]. SampEn was then calculated for the downsampled data for the AP and ML directions. SampEn conceptually quantifies the rate of information generation of the system, in this case, postural control. Furthermore, lower SampEn values are indicative of a more regular signal; meaning that the time series data are more repeatable. In contrast, higher SampEn values are more characteristic of an irregular signal, meaning that the time series data have a lower probability of occurring in the following data samples. In the literature, clinical populations usually have lower SampEn values than those who are healthy [28]. For example, a reduction in physiological function due to the natural aging or disease process will commonly result in lower SampEn values [20]. This is primarily characteristic of a less adaptable postural system that has decreased capacity to adjust the rate of information for a given strategy or task [20]. For clinicians, the calculation of SampEn is complex, but a MATLAB script is readily available to download from Physionet.org (https://physionet.org/content/sampen/1.0.0/).

SampEn was calculated as the negative natural logarithm of Ai, divided by Bi, where Ai was the number of similar vector lengths (m+1) that were within the tolerance range (r) of all possible (m+1) vectors and Bi was the total number of similar vector lengths at m that were within r at every possible vector length of m. This study used a vector length of 2 (m=2) and a tolerance range of 0.2 (*r*=0.2). SampEn was chosen over approximate entropy, as SampEn does not include not self-matching data, which can decrease the entropy value inadvertently [29].

### 2.4. Statistical analysis

Each dependent variable (SampEn ML and SampEn AP) was separately analyzed using a three-level linear mixed effects model or the multilevel model (MLM) using the “lme4” package [30] in R 3.5.1 [31]. The traditional repeated measures ANOVA was not used due to the three-level structure of the study design. Instead, a theory-driven MLM approach was where the modeling involved a three-way interaction model which was then compared to other simpler models for both dependent variables separately [32]. The significant interactions were confirmed through Satterthwaite’s degrees of freedom method implemented to the best fit model from lmerTest [33] and are reported as F values (significance).

Fixed effects were investigated for the observation attribute of the task (level 1: “Task” EO and EC), the time attribute (level 2: “Time” pre- and post-season), the participant attribute of group (level 3: “Group” RHI and CON), and the cross-level interaction between Task, Time, and Group. The reference category for Task was EO, Group was CON, and Time was pre-season. The level of significance was set at *P*<0.05.

## 3. Results

**Figure 1:**
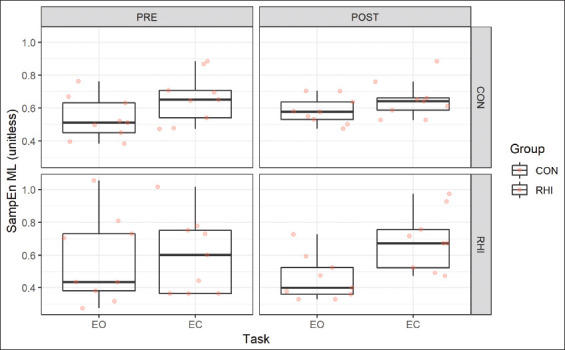
Raw box plot for sample entropy mediolateral as means for repetitive head impact and CON groups.

The MLM analyses for SampEn ML yielded a three-way interaction as the best fit model (Group by Task by Time), whereas for the SampEn AP, the MLM analyses yielded a two-way interaction (Task by Time) and a Group main effect as the best fit model. However, the post-hoc analyses using the Satterthwaite’s degrees of freedom for the best fit model did not show a significant effect at any level for SampEn AP. Therefore, the results for the best fit model for SampEn AP were not reported in the results. Figure 1 describes the raw plot where the mean SampEn ML for each participant is represented as box plots for both groups across the PRE and POST time points for each task.

**Figure 2:**
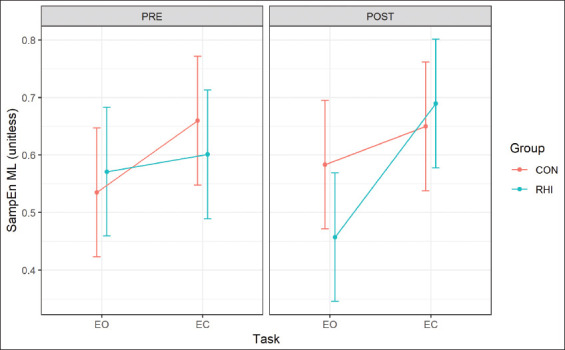
Multilevel model fit for sample entropy mediolateral as estimated marginal means for repetitive head impact and CON groups. Error bars are SE.

Table 2 describes the fixed and random effects used for the best fit model for SampEn ML which are verified and confirmed by the visual inspections in Figure 2. The Wald significance test revealed a significant Group by Task by Time interaction. The best fit model for SampEn ML had significant task main effect *F* (1, 48)=21.891, *P*<0.001 and a Group by Task by Time interaction *F* (1, 48)=7.219, *P*<0.01. The significant Task main effects were observed for the EC task. Specifically, the SampEn ML was significantly higher for EC compared to EO for both groups. This effect was specifically different between the time points where post-season has significantly higher SampEn for both groups than pre-season specifically for the EC task.

**Table 2:** Parameter estimates for the MLM regarding SampEn ML (unitless).

SampEn ML (unitless)				

Fixed effects	Est (SE)	Wald sig.	F (df)	SW sig.
(Intercept)	0.54 (0.06)	***		
Group (ref=CON)			0.156 (1)	
RHI	0.04 (0.08)			
Task (ref=EO)			21.892 (1)	***
EC	0.12 (0.05)	*		
Time (ref=pre-season)			0.018 (1)	
Post-season	0.05 (0.05)			
Group×Task	−0.09 (0.07)		0.541 (1)	
Group×Time	−0.16 (0.07)	*	0.433 (1)	
Task×Time	−0.06 (0.07)		2.202 (1)	
Group×Task×Time	0.26 (0.1)	**	7.219 (1)	**
Random effects	Var			
Intercept	0.02			
Residual	0.01			

*** *P*<0.001,

** *P*<0.01,

* *P*<0.05. Wald sig. uses a normal distribution whereas the SW sig. utilizes the Satterthwaite’s method for type III tests of fixed effects. Sample size=72 observations on 18 participants. SampEn: Sample entropy, ML: Mediolateral, RHI: Repetitive head impact, EO: Eyes open, EC: Eyes closed, AP: Anteroposterior

## 4. Discussion

The purpose of the current study was to investigate postural control changes (SampEn) in the ML and AP directions during quiet stance following a season of RHI among Division I football athletes. Specifically, we compared RHI athletes who received RHIs across the season but had no previously diagnosed sport related concussion to a group of non-RHI athletes (CON) with no diagnosed sport-related concussion. Our main finding indicated an effect of Task (EO and EC) and Time (pre- and post-season) for both groups (CON and RHI) in the ML direction for SampEn. However, we did not see such an effect in the AP direction. Our hypothesis was partially supported for the SampEn AP in the EO task between groups which is consistent with the previous findings [14,16]. However, the effect of Time and Task across the two seasons for SampEn in the ML direction was driven by the EC task in the RHI group. In addition, the EC task was significantly affected during post-season for RHI, while no changes were noted for the CON group. The novelty of the current results is driven by the SampEn in the ML directions and highlights the effect of the EC task that has not been reported in the literature.

At present, only one study has used and reported entropy (SampEn) changes in the AP direction with RHI as affected by sport season [14]. The authors only used an EO, quiet stance task and showed no significant differences pre- to post-season in SampEn in the AP direction. Vision is known to play an important role and influence postural control changes [24]. In addition, the elimination of visual feedback (in our case EC) has been reported to elicit significantly different center of pressure changes compared to the availability of visual feedback (in our case EO) [24]. While testing for postural control, the lack of visual feedback can decrease postural stability, which can indicate an effect of injury or pathology [21,34]. Furthermore, in the absence of visual feedback, there is a greater reliance on proprioception and vestibular responses; however, in the presence of vision, these responses are reduced [35]. In addition, EC tasks allow the observers to test sensory modalities that are not possible to test for which are present in the EO task. In addition to the task, concussion history has been noted to affect postural control function. In Murray *et al*. [14], the contact group included athletes with a history of concussion, while the control group did not have any history of concussion. Those with a history of concussions are reported to have significant postural control deficits during quiet and dynamic postural tasks [17-19]. Therefore, the novelty of the current study highlighted that across one athletic season, there was an increase in SampEn in the ML direction for EC that was driven by the RHI group.

In the current study, we observed an effect of Time and Task. This effect can be attributed to the sensory reweighting/shift in the dynamic physiological processes [36,37]. Our results indicated that at pre-season, there was an equal rate of information production from all the sensory systems that may have control EO and EC postural control (i.e., visual, vestibular, and proprioceptive systems). However, at post-season, there was a shift in the rate of information production indicated by an increased SampEn for EC compared to EO. This change may be due to reactive tuning within the physiological systems [36], which is changing the system’s dynamic equilibrium at certain time scales due to some external or internal stress, such as physical contact. This physical contact may influence proprioceptive and vestibular adaptation in posture control [38]. This supports the current findings that the RHI group’s SampEn ML changed from pre- to post-season, with an increase in the rate of information production in the EC task and a decrease in the rate of information production in the EO task. In addition, we speculate that the sensory reweighting may have been due to the effect of the sporting season or RHI; however at this time, it is unclear if the effect is solely the sporting season or RHI in the outcome variables.

The increased SampEn ML in the absence of a vision for both groups in the post-season is important to account for and has clinical implications for clinicians while assessing RHIs. Our findings highlight that the RHI group may have overcome a somatosensory deficit as an effect of the season seen as improved SampEn ML values between tasks. These findings should be further explored with RHI, but are promising to guide clinicians to incorporate tasks with deprived sensory inputs such as the absence of visual input. Evidence from Simoneau *et al*. [39] have established this idea of the importance of the input from the somatosensory system to be as important as the visual systems in clinical populations. In addition, the current study was able to report sensitive measures for both EO and EC postural control through SampEn that is not accounted for by traditional measurements.

The current study has a few limitations. First, we were not able to track the individual head impact frequencies across the season or from prior seasons in the RHI group. Therefore, we could not separate the RHI athletes into high- or low-impact groups. Finally, we were only able to test males in this study due to the nature of the sport selected.

## 5. Conclusions

The current study findings indicate center of pressure changes from pre- to post-season that shift the sensory reliance toward more proprioceptive and vestibular input and less toward contributions from the visual system, which was driven by the RHI than the CON group. However, this study did not find any differences between groups. Further research is needed to account for the relationship between the frequency of RHI and postural control variables. In addition, researchers should also account for these differences in multiple sports to account for gender- and sport-related effects on static postural control tasks.
